# A phase 3 clinical trial with ISIS-TTRRx, a 2nd-generation antisense oligonucleotide targeting transthyretin (TTR), for the treatment of TTR amyloid cardiomyopathy

**DOI:** 10.1186/1750-1172-10-S1-P8

**Published:** 2015-11-02

**Authors:** Helen Millns, Rito Bergemann, Elizabeth Ackermann, Brett Monia, Mary Ann Lukas

**Affiliations:** 1 GlaxoSmithKline, Clinical Statistics, SG1 2NY, Hertfordshire, UK; 2 GlaxoSmithKline, Rare Diseases, TW8 9GS, Middlesex, UK; 3 Isis Pharmaceuticals, Clinical Development, 92010, Carlsbad, USA; 4 GlaxoSmithKline, Clinical Development, 19112, Philadelphia, USA

## Background

Transthyretin (TTR) amyloidosis is a progressive and fatal systemic disorder caused by misfolded TTR monomers that cumulatively deposit in the heart, peripheral nerves and other organ systems. TTR Amyloidosis-associated cardiomyopathy (ATTR-CM), caused by TTR amyloid infiltration of the myocardium and conduction system, results in a restrictive cardiomyopathy associated with atrial arrhythmias, progressive heart failure with preserved ejection fraction in early phases, and leads to reduced life expectancy. ISIS-TTRRx is a first-in-class, 2nd-generation antisense oligonucleotide designed to produce substantial reductions in the levels of both mutant and wild type TTR produced from the liver.

## Methods

We plan to conduct a Phase 3 randomized, double-blind, placebo-controlled, global study designed to evaluate the efficacy and safety of ISIS-TTRRx in patients with ATTR-CM. The study will evaluate the differences observed between patients treated with placebo and with ISIS-TTRRx over 24 months evaluating several key endpoints. Serum biomarkers including circulating TTR levels, NT-pro-BNP and troponin, imaging endpoints including global longitudinal strain by echocardiography, clinical outcomes including mortality, transplants, cardiovascular hospitalization and arrhythmic events, and clinical status including New York Heart Association (NYHA) class, and the patient-reported instrument Kansas City Cardiomyopathy Questionnaire (KCCQ) will be evaluated. The trial is designed to enroll approximately 400 ATTR-CM patients with documented senile systemic amyloidosis (SSA) or documented familial amyloid cardiomyopathy (FAC), including theVal122Ile mutation and other TTR mutations, in addition to biopsy-proven amyloid deposits. Patients will be randomized 2:1, ISIS-TTRRx:placebo, with ISIS-TTRRx administered subcutaneously at 300 mg every other day for five days, then weekly for 24 months. The study will have 85% power to detect a difference between treatment groups in the final primary endpoint, with a significance level of p < 0.05. All patients completing the Phase 3 study will be eligible to enroll in a Phase 3 open-label extension study or otherwise receive access to ISIS-TTRRx. An academic Steering Committee will oversee trial conduct, an independent Clinical Event Committee will adjudicate study outcome endpoints, and an independent Data Safety Monitoring Committee will periodically review and evaluate accumulated study data for participant safety and, when appropriate, efficacy, and make recommendations to the sponsor and Steering Committee concerning the continuation, modification, or termination of the trial.

## Results and conclusions

Results of the trial are anticipated in 2019. The trial will establish the role of therapy with ISIS-TTRRx. in this patient population for whom effective treatments to alter the progression of the disease are needed.

